# Membrane Depth Measurements of E Protein by ^2^H ESEEM Spectroscopy in Lipid Bilayers

**DOI:** 10.3390/biophysica5040058

**Published:** 2025-11-26

**Authors:** Andrew K. Morris, Robert M. McCarrick, Gary A. Lorigan

**Affiliations:** Department of Chemistry and Biochemistry, Miami University, Oxford, OH 45056, USA

**Keywords:** EPR, ESEEM, envelope protein

## Abstract

A topological analysis was performed by taking ESEEM measurements of site-specifically labeled E protein from SARS-CoV-2. The intensity of deuterium modulation arising from either deuterated solvent or deuterated lipid acyl chains revealed exposure to solvent or the bilayer hydrophobic region. Spin-labeled lipids and soluble spin labels were used as points of comparison. The data indicate that spin labels placed along the transmembrane helix of the E protein showed close contact with lipid acyl chains, but also substantial contact with solvent, while those placed on the C-terminal domain showed substantial but lower exposure to lipid acyl chains, with comparable solvent exposure. The results support the view that the C-terminal domain is in contact with the bilayer surface.

## 1. Introduction

The envelope protein (E) is a critical component of the SARS-CoV-2 virus [1]. It is a 75-amino acid virally encoded protein which localizes to the endoplasmic reticulum–Golgi intermediate complex [2–4]. E is a viroporin, a class of membrane proteins within viral genomes that act as ion channels [5]. In addition, it plays an essential function in the formation of new virions by recruitment of other viral proteins [4,6]. E has also been shown to induce membrane curvature at the site of viral budding [7,8]. It is a single-pass transmembrane protein with an N-terminus on the lumenal side of the endoplasmic reticulum bilayer and C-terminus on the cytoplasmic side [2,9]. The transmembrane domain (TMD), which spans approximately residues 15 to 38, forms a homopentamer in its functional state as an ion channel [3,10]. The C-terminal domain (CTD) consists of two helices and an unstructured C-terminus, but its topology with respect to the membrane is less clear. A previous structure of the E protein from the closely related SARS virus obtained in LMPG detergent shows the CTD turning back inward into the membrane [11]. However, if this structure was in a lipid bilayer, it would place several charged residues within the hydrophobic region, and the model does not include the entire CTD. More recent structures of E in a native-like lipid mix were obtained, but the CTD is completely absent [12,13]. Reports have shown that the CTD of E may form a completely different beta sheet structure at a high protein to lipid ratio. However, no structure of the CTD in native lipids was unambiguously solved, and the exact manner by which this conformational switch is believed to occur is not clear [14,15]. To address some of the open questions regarding the topology of E, we applied the pulsed EPR electron spin echo envelope modulation (ESEEM) to spin-labeled E in liposomes.

Pulsed electron paramagnetic resonance methods, such as ESEEM, have become a valuable tool for biophysical studies. They offer unique advantages over other methodologies for certain applications because they are not limited by molecular size, require smaller sample quantities and concentrations, and allow for measurements to be made in a site-specific manner [16,17]. ESEEM spectroscopy is based on the coupling between electronic and nuclear spins, whose strength depends on the inverse 6th power of the distance between the coupled spins, as well as the orientation between their moments and the applied magnetic field. This sharp exponential decay with respect to distance means that all nuclei are effectively invisible except for those within a very close cutoff range, which for deuterium is approximately 0.8 nm [16–19].

For ESEEM experiments, the primary data are read as the decay of the intensity of the stimulated echo as a function of time. The modulation of this echo occurs due to coupling between the electronic spin being observed and spin-active nuclei when they are in close proximity. When the electron spin echo is collected, the primary decay can be fit and subtracted to show the oscillations induced by the nuclear couplings. When this is subject to a cross-term averaged fast Fourier transform, peaks emerge at the Larmor frequencies of these nuclei. The intensity of modulation is therefore indicative of the average number of nuclei within the spin envelope of the labeled site on the molecule in the sample [20,21].

**Figure 1.**
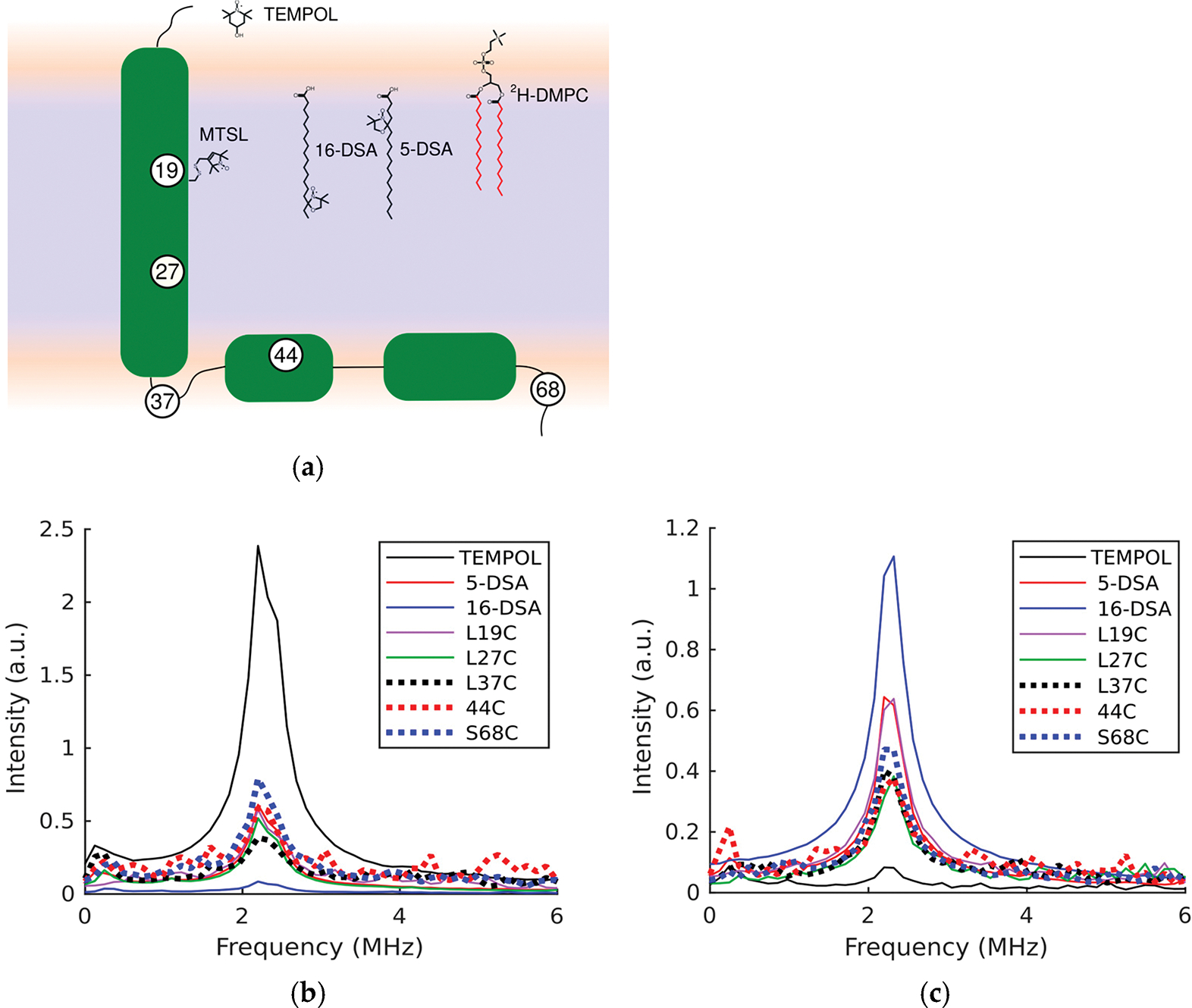
(**a**) Schematic of the E protein in green within the bilayer with labeled positions indicated, alongside spin labels used in this study as well the deuterated lipid DMPC with deuterated positions shown in red. (**b**) Fourier transform of ESEEM data with D_2_O. (**c**) Fourier transform of ESEEM data with d54-DMPC.

Our lab and others have utilized this to the effect of a molecular proximity sensor by looking for spin couplings with deuterium [22–24]. Site-directed spin labelling allows the targeted placement of nitroxides at any residue in a protein by introduction of a cysteine and attachment of the thiol-specific label MTSL [25]. Nitroxide spin labels can also be incorporated into fatty acids. When the doxyl moiety is placed at sequentially higher numbered carbons on the acyl chain, a means to query membrane properties in a depth-dependent manner is provided. This has been used extensively to provide a probe for specific depths within a bilayer and examine changes in solvent accessibility and order parameter with varying lipid composition, as well as in different nanodisc and copolymer systems [21,26–28]. Previous studies have shown there is an inverse dependence of the deuterium modulation intensity in spin-labeled lipids as a function of depth when liposomes are solvated in a deuterated buffer [29]. With D_2_O present, deuterium modulation indicates the extent of hydration, which is quite significant within the bilayer up to the 11-carbon position, and so is sensitive even for sites within the bilayer [21]. Conversely, if a non-deuterated solvent is used and instead lipids with deuterated acyl chains are added, then any labeled sites inside the membrane will show deuterium modulation to a similar extent, but labels outside the phosphate head groups should show very little modulation [24]. However, the latter experimental configuration with deuterated lipids has a much sharper delimiting region. The use of selective deuteration of either the lipids or solvent allows a way to determine if the attached spin label is in close proximity to either lipid acyl chains or aqueous solution, which affords information to membrane topology at specific sites along the protein or peptide sequence. The various spin labels used in this study are shown in Figure 1a, along with the labeled sites on E where MTSL was placed.

## 2. Materials and Methods

MTSL-labeled peptides were prepared by solid phase peptide synthesis on a Liberty Blue 2.0 automated synthesizer (CEM corporation, Matthews, NC, USA) at a 0.05 mmol scale using preconjugated Fmoc-Val-TGA resin. For deprotection, 10% piperidine solution at 90 °C for 1 min was used. Oxyma and diisopropyl carbodiimide were used for activation, and coupling was carried out with the addition of the FMOC-protected amino acid for 4 min at 90 °C. The coupling step was repeated for each cycle. A final deprotection was performed after the final coupling. After synthesis, peptides were cleaved from the resin in a solution of trichloroacetic acid with 2.5% *v*/*v* water, 1% *v*/*v* triisopropyl silane, and 2.5% *v*/*v* ethane dithiol. Crude peptide was precipitated by addition of diethyl ether, dried, and then purified by fractionation over a C18 reverse-phase column with a gradient of 70% acetonitrile/water to 95% n-propanol/water. Fractions were assessed for purity by electrospray ionization mass spectrometry. The desired fractions were labeled by reaction with a 10-fold molar excess of MTSL in a water/TFE mixture overnight. Excess label was removed by passing the reaction back over the reverse phase column and running the same gradient.

For D_2_O ESEEM samples, liposomes were prepared by the thin film method by mixing DPPC solution in chloroform with either MTSL-labeled peptide or doxyl lipids to a 1:250 molar ratio of spin-labeled species to DPPC. For measurements with TEMPOL, the same volume of lipid alone was used. Each was dried under a stream of nitrogen gas and then placed in a vacuum chamber overnight to remove residual solvent. The samples were then rehydrated with a buffer consisting of 30% *v*/*v* glycerol, 150 mM NaCl 50 mM HEPES, pH 8.0, prepared with D_2_O solvent. For the TEMPOL samples, the same buffer was used with 200 μM TEMPOL added. Each was vortexed vigorously and freeze-thawed 3 times, then sonicated in a water bath at 45 °C to have DPPC in the liquid crystalline phase. Samples were then placed at 4 °C for up to several days before transferring to 3 mm quartz tubes for measurement. Each sample was frozen in liquid nitrogen immediately prior to measurement on the instrument. For samples with deuterated lipid, the protocol was identical except that a 1:4 mol:mol mixture of d54-DMPC: DPPC was used in the preparation of thin films at the same overall protein to lipid ratio, and that the buffer used contained ^1^H H_2_O instead of D_2_O.

Three pulse ESEEM experiments were performed as previously described using a ELEXSYS E580 pulsed spectrometer (Bruker Biospin, Billerica, MA, USA) equipped with an EN4D resonator at 80 K at X band [30]. The length of the mixing time T was incremented in 12 ns steps beginning at 386 ns and τ was set to 216 ns. Echoes were normalized, and background decay fitted and subtracted, and the nuclear modulation was cross-term averaged fast Fourier transformed using MATLAB (v R2024a).

## 3. Results

Figure 1b shows FT ESEEM data of various spin labels in deuterated solvent. The water soluble nitroxide TEMPOL shows a very large peak at 2.28 MHz in the Fourier transform of the data after the primary unmodulated electronic echo is fitted and subtracted away, but in non-deuterated buffer with deuterated DMPC added into the lipid bilayer, the deuterium peak is almost completely absent. As another point of reference, 16-DSA, in which the spin label site is deep in the membrane core, shows essentially no modulation from D_2_O, but a much larger peak than any other spin label tested in the presence of deuterated lipids. Between these two extremes is 5-DSA, which shows an intermediate peak for ESEEM taken in both D_2_O and deuterated lipids. With these as benchmarks, the position of the spin labels when attached to proteins is put into context. Levels of modulation for MTSL placed on L19C, L37C, or L27C are slightly lower than that experienced by the 5-DSA when D_2_O is present. MTSL placed on 44C or S68C, which are in the CTD, shows slightly greater deuterium modulation than 5-DSA. Figure 1c shows that when deuterated lipids are used instead of D_2_O, the L19C label site shows modulation near the level of 5-DSA, while for L27C, S68C, and 44C, there is substantially less, but still much more than for the aqueous TEMPOL. Since water permeates into the membrane substantially up to the 11-carbon position for DPPC membranes, the modulation seen in Figure 1b indicates none of the sites tested on E are at the center of the hydrophobic core. L27C is further down the TMD helix, while L37C is expected to be right at the bilayer interface, and so these results are also consistent with the existing topological model.

## 4. Discussion

Although it has the lowest modulation, the L19C site might be expected to show nearly complete insensitivity to D_2_O similar to the 16-DSA standard since it lies in the center of the TMD. However, it is worth noting that the conformational flexibility of the MTSL label affords it a wide range of rotameric possibilities and, in turn, uncertainty in the position of the actual nitroxide paramagnetic center. It also has a noted tendency to pack against transmembrane helices rather than extend outward [31]. This is likely the source of the larger than expected modulation from D_2_O despite its position. This is also represented by its modulation from deuterated lipids, which is similar to that of 5-DSA as opposed to 16-DSA. Taken together, the extent of modulation at labeled sites in the TMD indicates that they are within the membrane, but that the relationship between expected depth and modulation intensity is not necessarily linear when D_2_O is used. This is consistent with the findings of Carmieli et al., who saw that the addition of peptide to a membrane of saturated lipids increases the penetration of water into the hydrophobic interior of a bilayer and flattens the expected response between membrane depth and modulation [24]. This also implies that the modulation intensity caused by deuterated lipids is a more reliable litmus test for discerning aqueous versus buried when probing a site than D_2_O-induced modulation, since it shows a greater response range and is not affected by the inherent effect of the peptide itself on the bilayer. This insight may prove useful for those wishing to use ESEEM as a topology measurement.

The most notable information about the E protein itself that is gleaned from these measurements pertains to sites in the CTD. 44C and S68C show substantial modulation from the deuterated lipid. Unlike water permeation into the bilayer, lipid acyl chains have a sharp boundary confining them to the hydrophobic core. Accordingly, the only way to see any substantial modulation from deuterated lipids is if these sites within the CTD are in proximity to the hydrophobic region. Unlike some topological models in which the CTD is completely solvated, this is more consistent with a model where CTD helices are adsorbed to the bilayer interface or buried at a shallow depth [15]. This may be closer to the published structure of the E pentamer from the original SARS virus taken in LMPG, in which the helices slant inward into the membrane, or the CTD helices may lie flat against the membrane [11]. Taken together, the data help to clarify the topology of the envelope protein and suggest it is in direct contact with the membrane throughout the CTD.

## 5. Conclusions

Five different sites along the primary sequence of the E protein were spin-labeled. The accessibility of the labeled sites to both D_2_O and d54-DMPC were measured by ESEEM for each site and compared against spin-label standards to gain site-specific information about the topology relative to the lipid bilayer. The results demonstrate that the CTD of E makes extensive contact with the membrane.

## Data Availability

All data have been deposited as follows: Morris, Andrew; McCarrick, Robert; Lorigan, Gary (2025), “SARS-CoV-2 Envelope ESEEM”, Mendeley Data, V1, https://doi.org/10.17632/fyd9xg5d97.1 (accessed on 1 October 2025).

## Abbreviations

EPR: electron paramagnetic resonance
ESEEM: electron spin echo envelope modulation
d54-DMPC: deuterated dimyristoylphosphatidylcholine
5-DSA: 5-doxyl stearic acid
16-DSA: 16-doxyl stearic acid
TEMPOL: 4-hydroxy-2,2,6,6-tetramethylpiperidin-1-oxyl
CTD: C-terminal domain
TMD: transmembrane Domain
FMOC: fluorenylmethoxycarbonyl
MTSL: S-(1-oxyl-2,2,5,5-tetramethyl-2,5-dihydro-1H-pyrrol-3-yl)methyl methanesulfonothioate
DPPC: dipalmitoylphosphatidylcholine
LMPG: lysomyristoylphosphatidylglycerol
TFE: trifluoroethanol
